# Estimating Muscle Mass Using D3-Creatine Dilution: A Narrative Review of Clinical Implications and Comparison With Other Methods

**DOI:** 10.1093/gerona/glad280

**Published:** 2023-12-22

**Authors:** Ana Paula Pagano, Julia Montenegro, Camila L P Oliveira, Nidhi Desai, M Cristina Gonzalez, Peggy M Cawthon, William J Evans, Carla M Prado

**Affiliations:** Human Nutrition Research Unit, Department of Agricultural, Food, and Nutritional Science, University of Alberta, Edmonton, Alberta, Canada; Women and Children’s Health Research Institute, Edmonton, Alberta, Canada; Human Nutrition Research Unit, Department of Agricultural, Food, and Nutritional Science, University of Alberta, Edmonton, Alberta, Canada; Human Nutrition Research Unit, Department of Agricultural, Food, and Nutritional Science, University of Alberta, Edmonton, Alberta, Canada; Human Nutrition Research Unit, Department of Agricultural, Food, and Nutritional Science, University of Alberta, Edmonton, Alberta, Canada; Faculty of Nursing, University of Calgary, Calgary, Alberta, Canada; Postgraduate Program in Nutrition and Food, Federal University of Pelotas, Pelotas, State of Rio Grande do Sul, Brazil; California Pacific Medical Center Research Institute, San Francisco, California, USA; Department of Epidemiology and Biostatistics, University of California San Francisco, San Francisco, California, USA; Department of Nutritional Sciences and Toxicology, University of California Berkeley, Berkeley, California, USA; Division of Geriatrics, Duke University School of Medicine, Durham, North Carolina, USA; Human Nutrition Research Unit, Department of Agricultural, Food, and Nutritional Science, University of Alberta, Edmonton, Alberta, Canada; Women and Children’s Health Research Institute, Edmonton, Alberta, Canada; (Medical Sciences Section)

**Keywords:** Body composition, Fat-free mass, Lean mass, Physical performance, Sarcopenia

## Abstract

**Background:**

The D3-creatine (D3-Cr) dilution method is of emerging interest for estimating total-body skeletal muscle mass. This review explores the association of muscle mass estimated via D3-Cr with various clinical outcomes and provides a summary of the literature comparing D3-Cr with other body composition techniques.

**Methods:**

A literature search was conducted on PubMed/MEDLINE and Web of Science for studies using D3-Cr to measure muscle in adult populations (ie, ≥18 years old) from inception until September 2023.

**Results:**

Out of the 23 included studies, 15 investigated the correlation between D3-Cr and clinical outcomes. More consistent associations were reported for mortality (100%, *n* = 2), mobility disability (100%; *n* = 5), falls and fractures (100%; *n* = 3), physical performance (63.3%; *n* = 11), muscle strength (44.4%; *n* = 9), and muscle composition (33.3%; *n* = 3). However, conflicting findings were also reported for such correlations. Among the 23 studies, 14 compared D3-Cr-estimated muscle with other body composition techniques, including magnetic resonance imaging (MRI) as a reference method. Strong and positive correlations were found between D3-Cr and MRI. Nonetheless, variations in muscle measurements were noted, with differences in D3-Cr values ranging from 0.62 kg lower to 13.47 kg higher compared to MRI.

**Conclusions:**

D3-Cr-estimated muscle mass may be a valuable predictor of clinical outcomes showing consistent associations with falls and fractures, mobility disability, and mortality. However, less consistent associations were found with muscle strength and composition, and physical performance. Although a strong correlation exists between D3-Cr-estimated muscle mass and MRI measurements, under- or overestimation may occur.

Muscle plays a crucial role in strength, mobility, balance, and whole-body metabolism (1). Both the quantity and composition (or “quality”) (2) of muscle are directly linked to an individual’s overall health status (1,3) such as surgical and postoperative complications, extended length of hospital stays, and shorter survival (3). The amount of muscle is of additional relevance in the context of aging due to age-related muscle loss, as approximately 50% of muscle mass is lost by the 8th and 9th decades of life (4). Such losses may be further accentuated by menopausal status due to unfavorable hormonal changes such as reduced estrogen levels (5). For older adults, muscle loss further relates to impaired muscle strength and physical performance, especially functional capacity (6,7). Preserving muscle strength and mobility is crucial during aging as it mitigates the risk of falls, injuries, and degenerative diseases. Consequently, it enhances overall health and quality of life during the aging process (8,9).

Accurate techniques are essential for assessing low muscle mass, a condition that may be concealed and present in individuals of any body size (3). Such assessment is crucial not only for diagnosing low muscle mass but also for identifying related conditions like malnutrition, sarcopenia, and cachexia (2,6,10–13). Several body composition techniques are used in research and clinical settings for assessing skeletal muscle mass including dual energy x-ray absorptiometry (DXA), magnetic resonance imaging (MRI), computed tomography (CT), bioimpedance analysis, and ultrasound (10,11,14–17). Although some techniques directly measure muscle mass, others assess related compartments such as lean soft tissue (LST) or fat-free mass (FFM), which include not only muscle but also other body components (17).

Recently, the D3-creatine (D3-Cr) dilution method has gained renewed attention as an alternative approach for estimating total body muscle mass and its contractile tissue components (18,19). This method determines muscle mass by directly measuring the total-body creatine (Cr) pool size through isotope dilution (20,21). The D3-Cr method offers several advantages, such as being noninvasive, safe, and causing minimal burden on the subject (19,22). The method involves administering a standardized dose of oral Cr-(methyl-D3) monohydrate (D3-Cr) to individuals and subsequently assessing the enrichment of urinary D3-creatinine (D3-Crn) (20,21) (Figure 1). The use of D3-Cr dilution was first tested in humans by Clark et al. (23). Since then, numerous original articles evaluated the D3-Cr-estimated muscle mass ability to associate with clinical outcomes such as falls and fractures, mobility/disability, and mortality, and tested its accuracy against different body composition techniques (24–30). However, a comprehensive review of the available evidence is still lacking. Given the growing interest in the D3-Cr dilution method, this narrative review aimed to delineate its clinical applications, particularly concerning D3-Cr-estimated muscle mass associates with components of sarcopenia and other clinical outcomes in both younger and older adults. Additionally, our secondary objective was to summarize the literature comparing D3-Cr with other body composition techniques.

**Figure 1.**
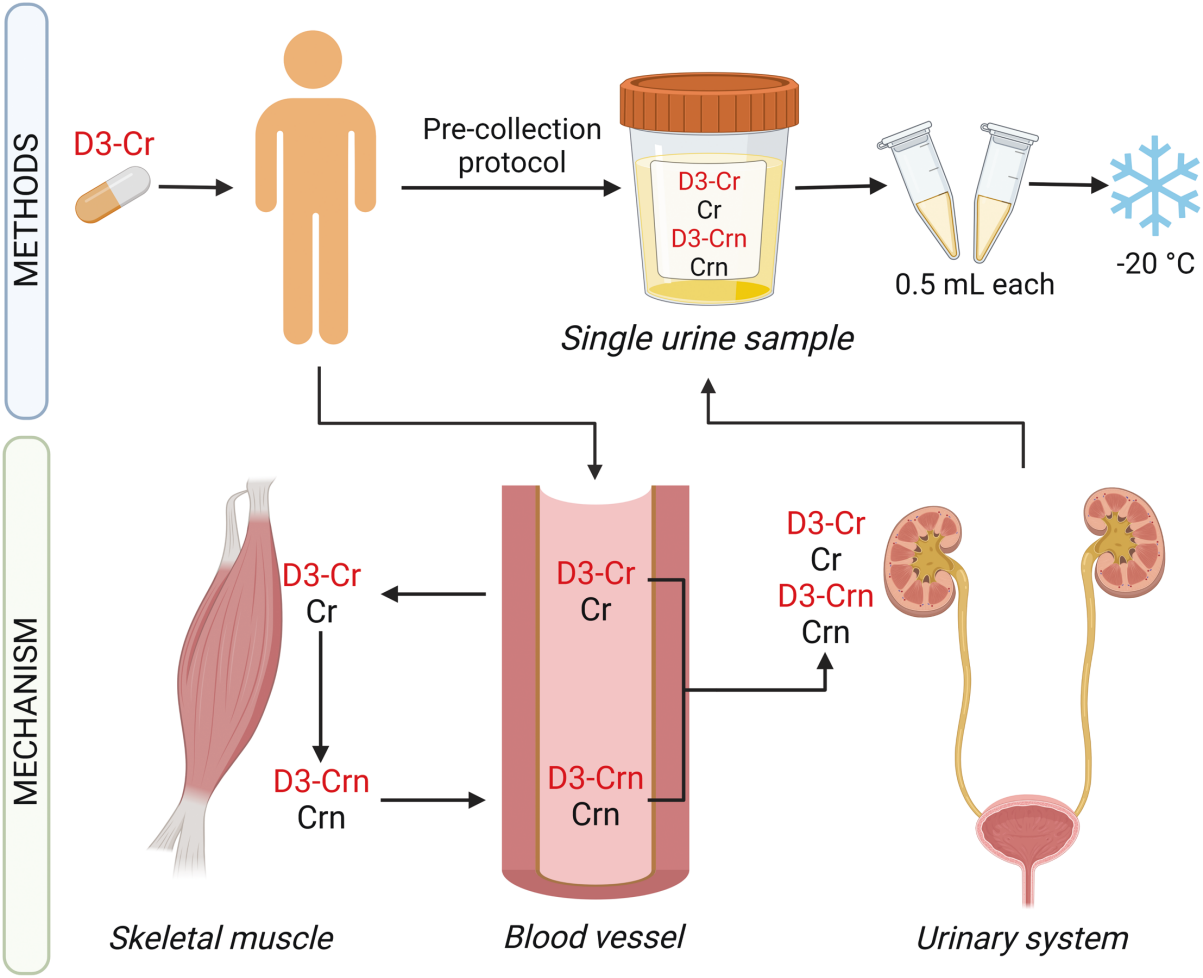
Methods and mechanisms on D3-Cr dilution method for estimating muscle mass. The D3-Cr dilution method is founded on the principle that about 95% of the total Cr pool in humans, which includes endogenous and exogenous sources, is located in skeletal muscle. Because Cr is converted irreversibly into Crn at a daily rate of roughly 2%, which is then completely excreted into urine, an oral dose of labeled Cr (ie, D3-Cr) can increase the isotopic concentration in urine. This enrichment enables the measurement of whole-body Cr pool size and subsequently the estimation skeletal muscle mass (23). To follow the recommended protocol for D3-Cr, an adult individual should ingest a 30 mg dose of D3-Cr, after which both exogenous D3-Cr, and exogenous and endogenous Cr are transported into muscle tissue and excreted into urine. To obtain a single urine sample, a precollection protocol must be followed, which includes collecting a sample between 48 and 96 hours after dosing, fasting for at least 8 hours, avoiding sources of Cr in the last meal before collection (because consuming such sources can alter the body’s Cr and Crn pool, potentially influencing the D3-Cr measurements). Provided that these requirements are followed, urine can be collected at any time, day or night, with no changes in results; however, the individual must refrain from collecting the first void in the morning. The collected urine sample may be processed into aliquots (0.5 mL) or using a filter-paper stick and stored at −20°C until analysis. For repeated measurements, a predose fasting urine sample is needed to correct for any residual D3-Cr from previous doses. Cr = creatine; Crn = creatinine; D3-Cr = D3-creatine.

## Method

### Literature Search

A literature search was performed in PubMed/MEDLINE and Web of Science from its inception until September 26, 2023. The search strategy consisted of 3 separate components, each involving key words related to “D3-creatine dilution method,” “body composition,” and “sarcopenia” individually (Supplementary Table 1). Key words in each component were linked using “OR” as a Boolean function, and the results of the 3 sections were combined by utilizing the “AND” Boolean in final search.

### Study Selection

The predefined study criteria targeted studies assessing body composition of adults using the D3-Cr dilution method for its predictive capabilities regarding clinical outcomes, and/or for its comparison against other body composition techniques. Clinical outcomes included functional outcomes, morbidity, and mortality. Studies were inputted into Covidence (https://app.covidence.org), which automatically remove duplicates. Two out of 4 reviewers individually screened titles and abstracts of retrieved studies, focusing on those that applied the D3-Cr dilution method to adults age 18 and above. Studies categorized as “eligible” were then thoroughly reviewed by 2 out of the 4 reviewers to confirm if they met the set criteria. Only primary research studies were considered. Nonoriginal articles, case studies, in vitro studies, studies with animal models, studies in pregnant or breastfeeding women, studies in children, reviews, and non-English publications were excluded. Any selection disagreements between reviewers were resolved by a third independent reviewer.

**Figure 2.**
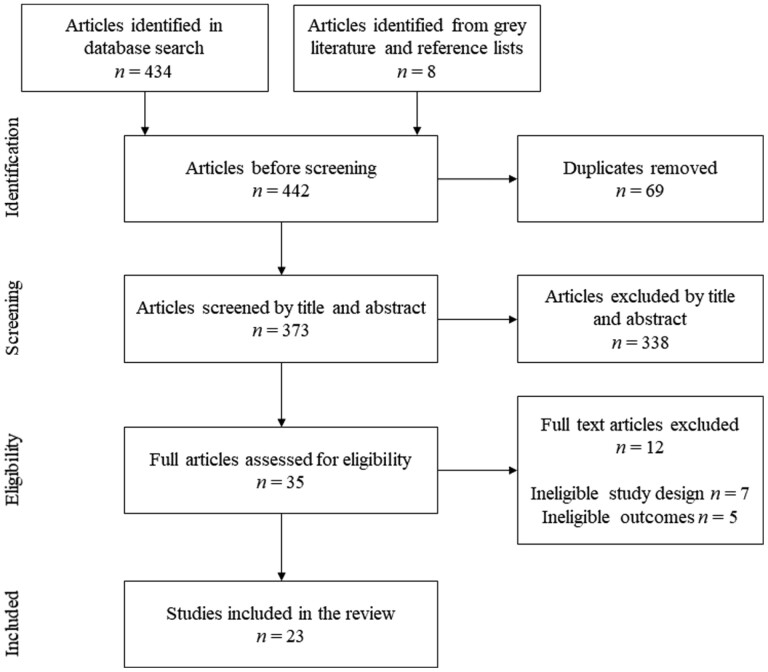
Flow chart describing the literature selection process of studies investigating D3-creatine dilution as a method for body composition assessment.

Reference lists of eligible articles were manually searched for additional studies that might have been missed during the electronic search; however, no additional titles emerged. A flow chart illustrating the literature selection process is shown in Figure 2.

### Data Extraction and Verification

A data extraction table was created to capture relevant information from included articles. An independent reviewer extracted the data, and a second reviewer verified the accuracy of extracted data. Disagreements were solved during a consensus meeting between the 2 reviewers. The extracted data included information considered relevant for the purpose of this review such as the association of muscle mass measurements by D3-Cr with components of sarcopenia (ie, muscle strength, muscle composition, and/or physical performance) and/or clinical outcomes (eg, mortality, mobility, and/or falls and fractures). Data comparing D3-Cr against other body composition techniques were also collected. Findings were not considered if the data were not shown. As this is a narrative review, a scale to evaluate the quality of studies was not included; in fact, the use of such tools may lack empirical support. Narrative reviews, unlike their systematic counterparts, do not have universally established protocols or standards (31). Moreover, even within systematic reviews and meta-analyses, the use of quality assessment tools for determining the inclusion or exclusion of articles is not advocated for, as it tends to introduce bias (32,33). Therefore, we did not evaluate the quality of included studies as there is no consensus, nor a requirement, that narrative reviews should incorporate them. Ethics approval is also not required for a narrative review.

### Data Synthesis and Analysis

Data from included articles were qualitatively synthesized and analyzed and findings were reported descriptively. D3-creatine ability to predict clinical outcomes and its comparison with other body composition techniques were reported according to the proportion of studies showing evidence of significant or nonsignificant findings, and the strength of such results (ie, weak, moderate, or strong). The evidence to report our findings was based on data provided by each included study and considering *p* values < .05, Bland–Altman analysis, confidence intervals that did not include 1.0, and strength of associations. The latter was categorized as very weak for correlation values between 0 and 0.19, weak for values between 0.2 and 0.39, moderate for values between 0.40 and 0.59, strong for values between 0.6 and 0.79, and very strong for values between 0.8 and 1.0.

## Results

### Characteristics of Included Studies

The literature search yielded 442 studies of which 23 studies met the inclusion criteria (23–30,34–48) (Figure 2). A summary of the main characteristics of the included studies is presented in Supplementary Table 2.

The majority of studies (78.3%) were conducted in the United States (23–25,27,28,30,34,35,37–43,45–47), and 10 out of the 23 studies were part of the Osteoporotic Fractures in Men (MrOS) cohort, a multicentric cohort investigating aging and osteoporosis across the United States (24,25,30,35,37–39,41,42,45). Most studies (74%) included only older adults (24–26,28,30,34–39,41–43,45–47). Three studies included both younger and older adults (13%) (23,27,40), and additional 3 included only younger adults (13%) (29,44,48). Sample size (range: 10 to 1 425 individuals) and participants’ mean age varied (range: 19.9 to 84.2 years) (23–30,34–48) (Supplementary Table 2). Study protocols differed in terms of the dose of D3-Cr administered to participants, collection of predose urine samples, time intervals for postdose urine sample collection, and number of samples collected (Supplementary Table 2). As only one study included D3-Cr-estimated muscle mass assessment on plasma sample in addition to urine sample (28), these data were not included.

All studies herein used the following equation to calculate Cr pool size (23–30,34–48):


$$\begin{array}{lllll} & Creatine\;pool\;size=\; & \;\left(131.1/134.1\right)x[amount\;of\;D3\text{-}Cr\;dosed\;\left(g\right)\;-\; & \;amount\;of\;D3\text{-}Cr\;excreted\;\left(g\right)]/\; & \left(mean\;steady\text{-}state\;D3\text{-}Cr\;enrichment\;ratio\right) \end{array}$$

Several studies (24,25,27,30,35,37–48) implemented a spillage correction to the aforementioned equation, using the algorithm developed by Shankaran et al. (40). Following the calculation of Cr pool size, muscle mass was estimated by dividing Cr pool size by 4.3 g/kg, a value signifying the concentration of Cr in whole-wet muscle mass (23). Notably, the research conducted by Morris-Paterson et al. (29) and Sagayama et al. (44) took into account a Cr concentration of 5.1 g/kg in whole-wet muscle and compared their findings to the standard 4.3 g/kg measure.

### Clinical Applications of D3-Creatine Dilution Method

**Figure 3.**
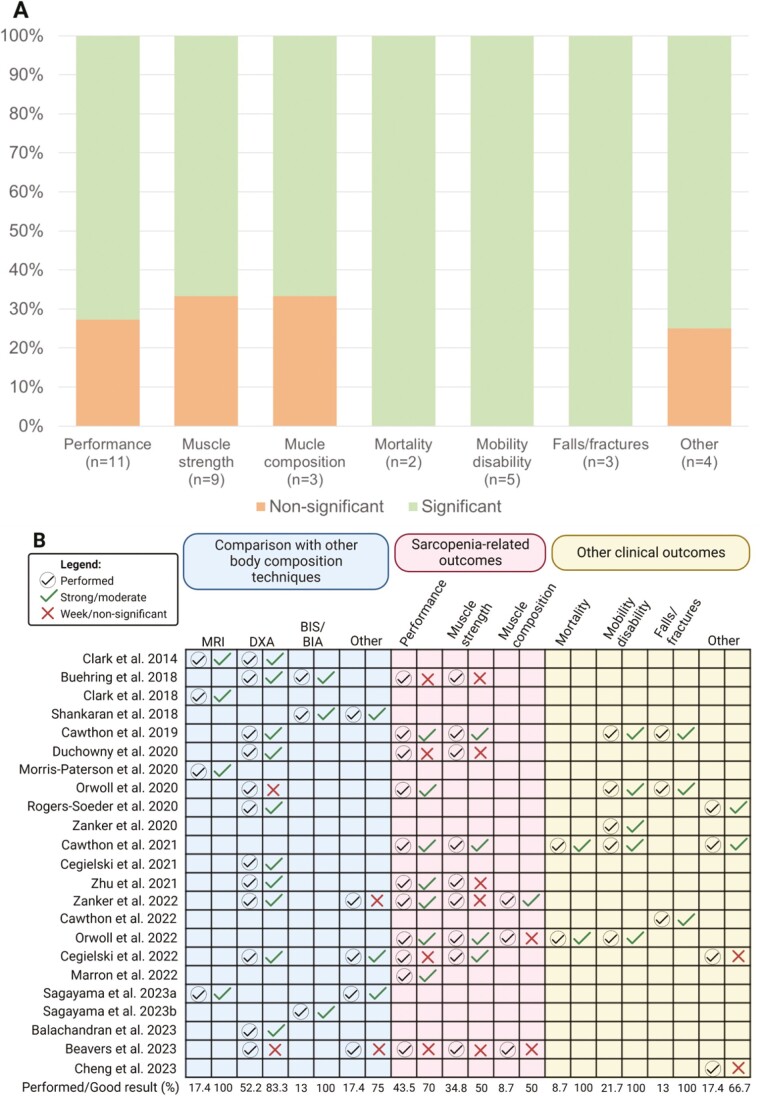
Summary of results from studies investigating the ability of D3-Cr to associate with clinical outcomes (A, B) and its comparison with other body composition techniques (B). (A) Percentage of studies demonstrating a significant or nonsignificant findings regarding clinical outcomes including physical performance, muscle strength, muscle composition (eg, muscle radiodensity or echo-intensity), mortality, mobility disability, falls and fractures, and others. The number of studies (*n*) exploring such associations is also shown. The direction of the significance was as expected: higher D3-Cr-estimated muscle related to higher physical performance, physical activity level, and muscle strength; while lower D3-Cr-estimated muscle associated with mortality, mobility disability, and falls/fractures. (B) Circled checkmarks indicate when the respective analysis was performed. Uncircled checkmarks indicate a strong/moderate correlation (*r* ≥ 0.4) or significant associations in regression models (*p* < .05). Crosses indicate week/no correlations (*r* < 0.4) or nonsignificant associations in regression models (*p* > .05). The percentage of studies that performed the test and found a significant result is shown at the bottom of its respective column. In the study by Buehring et al. (34), BIS total body water was compared against D3-Cr-estimated muscle. Other body composition assessments included: (1) 24-hour urine creatinine in the study by Shankaran et al. (40); (2) calf muscle area assessed by peripheral quantitative computed tomography in Zanker et al. (41); (3) vastus lateralis thickness assessed by ultrasound in Cegielski et al. (26); (4) 4-compartments model in Sagayama et al. (44); and (5) muscle in midtight and trunk assessed with computerized tomography in Beavers et al. (47). Other clinical outcomes include: (1) physical activity level according to quartiles of D3-Cr-estimated muscle mass in Rogers-Soeder et al. (39) and Cawthon et al. (24); (2) muscle protein synthesis per day, absolute synthesis rate, and muscle protein breakdown in the study by Cegielski et al. (26); and (3) risk of reduced relative dose intensity in Cheng et al. (27). All results refer to correlation and/or regression models, except in Rogers-Soeder et al. (39) that compared DXA appendicular lean soft tissue between quartiles of D3-Cr-estimated muscle mass, and in Cawthon et al. (24,25) who compared physical performance and strength between lower versus higher D3-Cr-estimated muscle mass. BIA/BIS = bioimpedance; D3-Cr = D3-creatine; DXA = dual-energy x-ray absorptiometry; MRI = magnetic resonance imaging; *n =* number of studies.

Fifteen (24–27,30,34,35,37–39,41–43,45,47) out of the 23 studies (23–30,34–48) investigated D3-Cr methods in association with at least one type of clinical outcomes, which included components of sarcopenia, mortality, mobility disability, falls and fractures, among others. A summary of results is presented in Figure 3. A detailed description of the main findings reported by each study is presented in Supplementary Table 3. Overall, D3-Cr was associated with these outcomes; however, significant and positive results were more frequently found for mortality (100%; *n* = 2), mobility disability (100%; *n* = 5), and falls and fractures (100%; *n* = 3), followed by physical performance (63.3%; *n* = 11), muscle strength (44.4%; *n* = 9), and muscle composition (33.3%; *n* = 3). Nonetheless, conflicting findings were also observed, and the number of studies investigating the association between D3-Cr and clinical outcomes is still limited (Figure 3B). Methodological heterogeneity was observed across studies, including variations in the physical performance tests used, the protocol followed to perform such tests, and the statistical analysis performed. For instance, to determine muscle strength, participants’ grip strength and/or lower extremity power and force were assessed (24–26,34,37,38,41,43,47). Physical performance tests included jump power, Timed Up & Go Test, gait speed, repeated chair stand, and balance assessments (24–26,30,34,37,38,41,43,45,47). Additionally, the use of adjustments varied among studies and played a critical role in certain results. For example, Duchowny et al. found a moderate and positive correlation between D3-Cr-estimated muscle changes adjusted for weight and walking speed changes, while this correlation was not observed without weight adjustment; however, such correlations were superior to that found by DXA (37). Similarly, Orwoll et al. reported the same association when applying a linear regression model adjusted for multiple factors, including weight and height (38) (Supplementary Table 3).

### D3-Creatine Dilution Method Versus Other Body Composition Techniques

Out of the 23 included studies (23–30,34–48), 14 compared muscle mass measured by D3-Cr method against its correspondent compartment measured by other body composition techniques (eg, LST or FFM) (23,25,26,28–30,34,36,37,39–41,43,44,46–48). The performance of the D3-Cr method in assessing muscle mass compared to each body composition technique is presented in Figure 3B. Equipment specifications, when available, are shown in Supplementary Table 2.

#### Magnetic resonance imaging

Magnetic resonance imaging assesses body composition at the tissue-organ level, allowing muscle mass to be measured (17). The method estimates muscle volume and converts it to muscle mass under the assumption that skeletal muscle has a density of 1.04 kg/L (49). Under normal conditions, the main determinants of muscle density are fluid (water; ~80%) and protein (~15%) (50). Although errors during conversion may exist, their impact would be minimum because muscle water varies up and down by a small range (51). Similarly, the inclusion of lipid content in muscle volume due to intermuscular adipose tissue being captured during manual segmentation may occur (52). Additionally, the MRI analysis may not effectively capture small muscle groups, such as those in the orbit (53) or toes (54). However, these issues would not lead to major errors and would most likely result in MRI measurements that are marginally lower than actual values, rather than significantly skewing the numbers. Notably, even the most “precise” method for measuring total body skeletal muscle mass, which involves the direct dissection of cadavers, is not immune to similar types of errors (55).

The D3-Cr dilution method was compared against MRI in 4 (23,28,29,44) out of the 23 studies (23–30,34–48), and a significant and positive correlation between methods was observed in all studies, varying from high (*r* = 0.840) (44) to very high (*r* = 0.913) (28) (Supplementary Table 3). However, agreement between D3-Cr and MRI varied depending on the assumption of Cr concentration in whole-wet muscle. For example, the range varied from 0.62 kg lower to 13.47 kg higher than muscle mass measured by MRI for a Cr concentration of 4.3 g/kg, and from 5.35 kg lower to 5.85 kg higher than muscle mass measured by MRI for a Cr concentration of 5.1 g/kg (29). Notably, variability between measurements was greater in males than females in the study by Clark et al. (23), while the opposite was observed in the study by Morris-Paterson et al. (29). A summary of main outcomes from included studies is presented in Supplementary Table 3.

#### Dual energy x-ray absorptiometry

Lean soft tissue, formerly termed lean body mass, comprises body water, total body protein, carbohydrates, nonfat lipids, and soft tissue mineral (17). Although muscle is part of the LST compartment, LST also encompasses other tissues. Therefore, DXA-assessed LST is higher than D3-Cr-estimated muscle mass (30). Lean soft tissue from arms and legs is referred to as appendicular LST (ALST) (17) and is considered to better estimate muscle mass. This is because 75% of the body’s skeletal muscle is located in the arms and legs, which are less affected by organs in the trunk and head, and ALST is mainly muscle (≈76%) (56).

Twelve (23,25,26,30,34,36,37,39,41,43,46,47) of the 23 studies (23–30,34–48) compared D3-Cr against DXA (Figure 3B), but methodologies and findings reported among studies were variable. Specifically, studies investigated D3-Cr compared to DXA LST and/or ALST and/or changes in these compartments (23,25,26,30,34,36,37,39,41,43,46,47). Although most studies reported a positive correlation between D3-Cr-estimated muscle and DXA LST, varying from moderate (*r* = 0.50) (43) to high (*r* = 0.871) (26), Cegielski et al. found a moderate and significant correlation when using DXA ALST (*r* = 0.69) but not LST (36) (Supplementary Table 3). Some authors adopted different adjustment approaches (weight, height^2^, or body mass index [BMI]) to compare D3-Cr dilution with DXA, and conclusions regarding the applicability of such adjustments varied. Main findings regarding comparisons between D3-Cr and DXA, and the use of adjustments are presented in Supplementary Table 3.

#### Other body composition techniques

Other body composition techniques, such as bioimpedance spectroscopy (BIS) or multifrequency bioimpedance (BIA) were used in 2 (34,40) and 1 (48) out of the 23 studies (23–30,34–48). Fat-free mass can be assessed by BIS and corresponds to the sum of LST and bone mineral components (17). Shankaran et al. reported a positive and strong correlation between D3-Cr-estimated muscle mass and BIS FFM (*r* = 0.893) (40) (Supplementary Table 3). Moreover, mean FFM was 42 ± 7 kg in females and 62 ± 10 kg in males, whereas muscle mass measured by D3-Cr dilution method was 23 ± 4 kg in females and 37 ± 10 kg in males (40). Similar to LST, a large difference in kg measurements between different methods was expected (ie, BIS FFM vs D3-Cr-estimated muscle mass), as muscle mass is only one of the FFM components. The same study additionally compared D3-Cr-estimated muscle to 24-hour urine creatinine (Crn) and reported a strong and positive correlation between methods (*r* = 0.858) (40). Conversely, Zanker et al. assessed calf muscle area using peripheral quantitative CT and found a weak correlation (*r* = 0.28) compared to D3-Cr-estimated muscle (41). Cegielski et al. assessed vastus lateralis thickness using ultrasound, and a weak correlation (*r* = 0.36) was found (26). Sagayama et al. compared D3-Cr-estimated muscle to FFM from a 4-compartment model that included body mass, body volume, total body water, and bone mineral content and found a significant and high positive correlation (*r* = 0.859) between methods (44). Finally, Beavers et al. found no association between changes in D3-Cr-estimated muscle compared to changes in muscle measured by CT (47) (Supplementary Table 3).

## Discussion

This is the first review summarizing the association between D3-Cr and clinical outcomes and its comparison with other body composition techniques. Although previous reviews on D3-Cr were published; the focus was on describing its methodology, strengths, and limitations, or its applicability to investigate low muscle mass (18,57). Our findings suggest that D3-Cr-estimated muscle measures could potentially associate with clinical outcomes in a cohort of both younger and older adults, regardless of chronic health conditions. Results indicated a superior association of D3-Cr-estimated muscle mass with mortality, mobility disability, falls and fractures, and components of sarcopenia such as muscle strength and physical performance, compared to other body composition techniques, mainly DXA. However, due to the limited number of studies exploring this association, our conclusions should be interpreted cautiously.

Our findings reveal a stronger correlation between D3-Cr dilution method and MRI as a reference method for measuring muscle mass, compared to other body composition techniques, such as DXA and BIS. Discrepancies in the agreement between D3-Cr and MRI muscle measurements were reported as they depend on the assumption of Cr concentration in whole-wet muscle, although such assumptions would not affect the correlations. Thus, D3-Cr dilution method may still over- or underestimate muscle mass compared to MRI, and the concentration of Cr assumed in whole-wet muscle (ie, 4.3 vs 5.1 g/kg) seems to play a role in such discrepancies, as also supported by a recent review where numbers varied from 3.8 to 5.4 g/kg (19). Therefore, we hypothesize if different Cr concentrations should be considered based on population characteristics such as sex, age, and lifestyle factors such as diet and physical activity. Creatine concentration may be higher for males (58), and younger adults (59), and it tends to be lower in vegetarians and vegans as opposed to those on an omnivorous diet (19). Additionally, Cr concentration may differ in those with more active lifestyle (eg, some athletes may have higher Cr concentration than sedentary individuals (60)). Engaging in physical activity can lead to increased muscle mass. This growth often results in a higher Cr concentration in the muscle, as muscle size primarily determines the Cr pool size and its production (60). A previous review suggested that the practice of certain types of physical exercise alone or combined with Cr supplementation may increase muscle strength. However, the same review also highlighted some conflicting results (61). Although the evidence related to whether Cr supplementation increases or not water retention is conflicting (62), hydration status should not affect the D3-Cr dilution method as it detects the enrichment ratio between the tracer and endogenous Crn (23). Thus, the D3-Cr dilution method seems to be suitable for those with conditions associated with water imbalance (23) such as kidney disease. However, we did not find studies assessing whether water imbalance alters D3-Cr concentration in the muscle.

### D3-Cr and Clinical Outcomes

Although most studies reported strong or moderate correlations between D3-Cr and sarcopenia components or clinical outcomes, various tests and methodological approaches were used. Although most authors did not adjust the muscle mass (or its correspondent compartment) for analysis, others used different adjustment factors, such as weight and height^2^, with conflicting findings reported. McCarthy et al. discussed the advantages of adjusted versus absolute pool size approaches to measure D3-Cr-estimated muscle in their review (19). The authors reported that height^2^ adjustment can improve the reflection of muscle measures between individual differences in body size, thereby enhancing the distinction between muscle mass and contractile mass. Moreover, this approach may correct potential errors associated with different assumptions of Cr in whole-wet muscle mass (ie, 4.3 vs 5.1 g/kg). The authors further noted that adjustments may convert Cr pool size to muscle cell protein, which is not only associated with contractile function but also supports the development of sophisticated body composition models as muscle cell protein is a molecular-level body composition component (19). Thus, the use of adjustments may provide a superior interpretation of findings when analyzing correlations between D3-Cr and clinical outcomes.

### D3-Cr Comparison With Body Composition Techniques

Studies included in this review that compared D3-Cr against other body composition techniques demonstrated a positive correlation between D3-Cr-estimated muscle and DXA LST or ALST (23,25,26,34,36,43,46). Moreover, changes in the compartment measured by D3-Cr were also correlated with changes in the compartment measured by DXA (37). However, conflicting findings were reported when adjustments were made compared to absolute values. For instance, adjusting muscle mass (or LST or ALST) by weight, BMI, or height^2^ appeared to improve the correlation with D3-Cr measurements when D3-Cr was also adjusted by the same variables (25,37,41,43). Nonetheless, opposing findings regarding adjustments were also reported (25,37,41,43). Therefore, the use of variables such as weight, BMI, or height^2^ to correct for potential variations in muscle mass measurement (or its correspondent compartment) due to one’s body size should be further explored. D3-creatine also showed a positive correlation with DXA and BIA/BIS measures, but BIA/BIS FFM correlation was stronger compared to those from DXA. However, this comparison was limited to 2 studies (40,48), and therefore findings should be interpreted with caution. Similarly, limited number of studies found promising results when investigating D3-Cr-estimated muscle compared to additional body composition techniques such as 24-hour urine Crn (40), ultrasound (26), CT (41,47), and 4-compartment model (44). These results should also be interpreted carefully.

Identifying low muscle mass or muscle losses is crucial, particularly as muscle deterioration becomes more pronounced with aging (4), which may lead to a detrimental impact on overall health and quality of life of older adults (8,9). The availability of techniques to accurately detect low muscle mass or muscle losses can substantially improve health assessment of these individuals (2). Such detection can contribute to the development of targeted nutrition and physical activity recommendations, aiming to prevent muscle losses and improve health outcomes (2). Recently, there has been renewed interest in the D3-Cr dilution method as a potential technique for estimating muscle mass (19). However, the number of studies investigating its association with clinical outcomes and/or comparing its measurements against other body composition techniques, including reference methods and bedside tools, is still limited. Although this review included 23 studies (23–30,34–48), only 17 compared D3-Cr-estimated muscle against other body composition techniques (23,25,26,28–30,34,36,37,39–41,43,44,46–48), with 10 of them using data from the same male population—the MrOS cohort (24,25,30,35,37–39,41,42,45).

### D3-Cr in Research and Clinical Settings and Its Limitations

Currently, D3-Cr is more suitable for research settings as it relies on the use of high-performance liquid chromatography to quantify D3-Cr, D3-Crn, Cr, and Crn in urine (23), which is a sophisticated analysis not readily available in clinical settings. However, this is arguably an analysis that can be made available in several research and commercial laboratories. Although the range of agreement between D3-Cr and MRI was large, D3-Cr was found to have a strong and positive correlation with MRI muscle mass (23,28,29,44), and is a more cost-effective and feasible technique compared to reference methods such as MRI and CT, which are more costly and difficult to perform in large cohorts, frails individuals, children, or infants (2).

Further research should clarify the value of D3-Cr method to estimate muscle mass. For instance, it is yet unclear whether different Cr concentrations in the muscle should be considered for populations with different characteristics such as age, sex, lifestyle (including physical activity and diet), and body hydration as these could affect the measurement. As mentioned previously, we did not find studies assessing whether body hydration (ie, the presence of water imbalance) would alter the D3-Cr concentration in the muscle, and the potential impact of the findings. The accuracy between D3-Cr dilution method and a reference method has been previously analyzed based on agreement or correlation (23,28,29,44); however, to our knowledge, test-retest reliability studies have not been performed to date. Studies of this nature are essential to ascertain the consistency of the D3-Cr method in reporting similar results within the producing similar results within the same subject, over a specific period, as well as its applicability for longitudinal studies.

Currently, implementing the D3-Cr dilution method in clinical settings may not be possible due to its high costs and time to obtain results compared to what is currently viable in this setting. The costs may include purchasing and encapsulating isotopes, shipping of samples to specialized laboratories, and analyzing the samples. Additional challenges include the need of relying on patients’ adherence to adequately follow instructions for collecting a urine sample at their home on a specific date and time. If the sample is to be collected at a medical center, the individual will have the burden of traveling to the facility for an extra day for such a procedure. However, many of these challenges are similar to those of other body composition techniques and can be substantially minimized with ongoing developments in the field.

### Study Limitations

Our study is not absent of limitations. For instance, in spite of using “sarcopenia” as one of our search terms, we were unable to find studies purposefully including people diagnosed with sarcopenia or comparing them to those without this condition. Nonetheless, some patients in the reviewed studies, particularly those involving older adults, might have sarcopenia. Moreover, we observed heterogeneity among reviewed studies in aspects such as the age of participants, reported clinical outcomes, reference body composition techniques, and data analysis methods. This underscores the need for cautious interpretation of our findings. Finally, despite the emerging interest in the D3-Cr method, the limited number of published studies restricts our ability to provide a more in-depth interpretation of the available evidence. It is also worth noting that most of the studies we included originated from the same MrOS cohort in the United States (24,25,30,35,37–39,41,42,45).

## Future Directions

The use and practicality of D3-Cr in associating with clinical outcomes and muscle mass estimations in various populations require further investigation to grasp its broader application in different settings. Future studies should focus on standardizing the use of different Cr concentrations in whole-wet muscle, as this can influence its comparability to other body composition techniques, accounting for factors such as age, sex, physical activity levels, and diet. Moreover, test-retest reliability studies are needed to improve our understanding of the D3-Cr method’s accuracy.

Additional research is also needed to develop a standardized protocol for D3-Cr use. Currently, a minimum of 30 mg of isotopes is recommended for D3-Cr-estimated muscle analyses (23). Although higher doses have been tested (23), lower doses have not and warrant further studies to determine their suitability for measurements, which may reduce costs. Standardization is required for predose urine sample collection, postdose urine sample collection timing, and the number of urine samples to be collected across studies. Based on existing studies (23–26,28–30,34–45) and our experience with the method, we propose a protocol for using the D3-Cr dilution method, summarized in Figure 1.

## Conclusion

Our findings suggest that the D3-Cr can be used to estimate muscle mass, which is associated with clinical outcomes, including mortality. However, due to the limited number of studies and their inherent heterogeneity, these results warrant cautious interpretation. Although D3-Cr shows promise as a potential alternative to MRI for body composition assessment, disparities between methods persist. Further research is crucial to validate its precision and identify measures to minimize errors. The feasibility of D3-Cr in clinical settings remains uncertain, given its present limitations. However, future standardizing of the protocol and increased availability may address these challenges.

## Supplementary Material

glad280_suppl_Supplementary_Tables_S1-S3

## Data Availability

Figures 1 and 3B were created with BioRender.com
