# Supplementary material for: Estimating Muscle Mass Using D3-Creatine Dilution: A Narrative Review of Clinical Implications and Comparison With Other Methods
Source: J Gerontol A Biol Sci Med Sci. 2023 Dec 22;79(4):glad280. doi: 10.1093/gerona/glad280 (PMC10959434; doi:10.1093/gerona/glad280)
Supplement: glad280_suppl_Supplementary_Tables_S1-S3 [file glad280_suppl_supplementary_tables_s1-s3.pdf]

Supplemental Material

eTable 1. Search strategy performed in Ovid MEDLINE, and Web of Science

eTable 2. Summary of main characteristics and methodology from studies included in this review

eTable3. Summary of main findings from studies included in this review comparing D3-creatine dilution with other body composition techniques to measure muscle mass and/or its correspondent compartment as well as its ability to associate with clinical outcomes

eTable 1. Search strategy performed in Ovid MEDLINE, and Web of Science

**Database**	**Search Strategy**
MEDLINE<br><br>Ovid MEDLINE From inception until January 17, 2023	1. exp Body Composition/<br>2. (((fat-free or fat or muscle or bone or lean or lean soft or intramuscular adipose or adipose) adj2 (mass or tissue)) or total body water or bone mineral density or bone mineral content or body cell mass or skeletal muscle).mp.<br>3. 1 or 2<br>4. Exp Sarcopenia/<br>5. (cachexia or sarcopenic obesity or myosteatosis).mp.<br>6. 4 or 5<br>7. ((D3 or methyl-D3 or deuterated) adj2 (creatine or dilution)).mp.<br>8. (D3 creatine dilution method or Creatine methyl-D3 dilution or Deuterated creatine dilution or Creatine dilution or Creatine dilution method).mp.<br>9. 7 or 8<br>10. 3 or 6<br>11. 9 and 10
Web of Science	1. ((TS=(fat-free or fat or muscle or bone or lean or lean soft or intramuscular adipose or adipose near/2 mass or tissue)) OR TS=(total body water or bone mineral density or bone mineral content or body cell mass or skeletal muscle)) OR TS=(Body Composition)

Database	Search Strategy
From inception until January 17, 2023	2. TS=(Sarcopenia or cachexia or sarcopenic obesity or myosteatosis)<br>3. #2 OR #1<br>4. (((((TS=(D3 creatine dilution method)) OR TS=(Creatine methyl-D3 dilution)) OR TS=(Deuterated creatine dilution)) OR TS=(Creatine dilution)) OR TS=(Creatine dilution method)) OR TS=(D3 creatine)<br>5. #4 AND #3

eTable 2. Summary of main characteristics and methodology from studies included in this review

Author, year	Location	Population	Sample size (n)	Mean age ± SD (y)	D3-Cr dose	Protocol for urine collection	Additional body composition technique
**Clark et al. 2014 (1)**	United States	Healthy subjects were divided into four groups: 1) Young men (19 –30 y); 2) Older men (70 – 84 y); 3) Postmenopausal women (51– 62 y);	33 (13 young men, 6 older men, 9 postmenopausal women, and 5 older women)	Total: 51 ± 23<br>Young men: 23 ± 3.6<br>Older men: 75 ± 4.7<br>Postmenopausal women: 57 ± 3.4	100 mg for 7 young men and 4 postmenopausal women;<br>60 mg for 6 young men;<br>30 mg for 6 postmenopausal women	Pre-dose urine sample collected: yes<br>Post-dose urine sample collected: from 0h to 120h post-dose (at 5 time-points per day, with 4-h intervals between each collection)	MRI (equipment specification N/A)<br>iDXA (GE Lunar iDXA)

Author, year	Location	Population	Sample size (n)	Mean age ± SD (y)	D3-Cr dose	Protocol for urine collection	Additional body composition technique
		Older women (70 – 84 y)		Older women: 76 ± 3.7	and for all older men and women		
**Buehring et al. 2018 (2)**	Not specified (may assume United States based on author's affiliation)	Ambulatory community-dwelling men and women aged ≥70 y, with no recent surgery or injury	112 (89 women and 23 men)	Total: 80.6 ± 6<br><br>Women: 80 ± 5.9<br><br>Men: 83.2 ± 25.4	Not specified	Pre-dose urine sample collected: not specified<br><br>Post-dose urine sample collected: random sample collected at 4h and a morning	iDXA (GE Lunar iDXA)<br>BIS (ImpediMed SFB7)

Author, year	Location	Population	Sample size (n)	Mean age ± SD (y)	D3-Cr dose	Protocol for urine collection	Additional body composition technique
						collection 48-72hpost-dose	
**Clark et al. 2018 (3)**	United States	Older individuals were divided into three groups:<br>1) Healthy older men;<br>2) Healthy post-menopausal women;<br>3) Men with chronic	18 (10 healthy older men, 4 healthy postmenopausal women, 4 men with chronic health conditions)	Total: 73.3 ± 5.8<br><br>Healthy: 74.4 ± 4.8<br><br>Congestive heart failure: 75 ± 1.4	Single dose of 30 mg	Pre-dose urine sample collected: not specified<br><br>Post-dose urine sample collected: from 4h to 120h post-dose (with 4–8h intervals between each collection)	MRI (equipment specification N/A)

Author, year	Location	Population	Sample size (n)	Mean age ± SD (y)	D3-Cr dose	Protocol for urine collection	Additional body composition technique
		health conditions		Chronic obstructive pulmonary disease: 64.5 ± 9.2			
**Shankaran et al. 2018 (4)**	United States	Men and women across a large range of ages (18–100 y)	36 (19 women and 17 men)	Women: 44 ± 16<br><br>Men: 51 ± 18	Single dose of 60 mg	Pre-dose urine sample collected: yes<br><br>Post-dose urine sample collected: second void of fasting morning	BIS (ImpediMed SFB7)

Author, year	Location	Population	Sample size (n)	Mean age ± SD (y)	D3-Cr dose	Protocol for urine collection	Additional body composition technique
						urine during 4 days (i.e., up to 96 h post-dose). The total 24h urine of 3 days (post-dose) was also collected.	
**Cawthon et al. 2019 (5)**	United States	Ambulatory community-dwelling men aged ≥65 y without bilateral hip replacements	1,382	N/A[a]	Single dose of 30 mg	Pre-dose urine sample collected: not specified<br><br>Post-dose urine sample collected:	DXA (Hologic 4500 scanners, Waltham, MA)

**Author, year**	**Location**	**Population**	**Sample size (n)**	**Mean age ± SD (y)**	**D3-Cr dose**	**Protocol for urine collection**	**Additional body composition technique**
						morning sample, 72–144h post-dose	
**Duchowny et al. 2020 (6)**	United States	Ambulatory community-dwelling men aged ≥65 y without bilateral hip replacements	40	83.3 ± 3.9	Single dose of 30 mg	Pre-dose urine sample collected: not specified<br><br>Post-dose urine sample collected: fasting, single-void urine sample, 72–144h post-dose	DXA (Hologic 4500 scanners, Waltham, MA)

Author, year	Location	Population	Sample size (n)	Mean age ± SD (y)	D3-Cr dose	Protocol for urine collection	Additional body composition technique
**Morris-Patterson et al. 2020 (7)**	United Kingdom	Athletes who specialized in sprint canoe over 200, 500, and 1000 m distances in single and double boats	20	N/A[b]	Single dose of 60 mg (divided in two 30 mg capsules)	Pre-dose urine sample collected: yes<br><br>Post-dose urine sample collected: total urine output from baseline to 120h post-dose	MRI (Siemens Tim Trio 3T, Siemens Healthcare, Erlangen Germany)
**Orwoll et al. 2020 (8)**	United States	Ambulatory community-dwelling men aged ≥65 y without	1,376	84.2 ± 4	Single dose of 30 mg[c]	Pre-dose urine sample collected: not specified	DXA (Hologic 4500 scanners, Waltham, MA)

Author, year	Location	Population	Sample size (n)	Mean age ± SD (y)	D3-Cr dose	Protocol for urine collection	Additional body composition technique
		bilateral hip replacements				Post-dose urine sample collected: fasting, morning urine sample, 72–144h post-dose	
**Rogers-Soeder et al. 2020 (9)**	United States	Ambulatory community-dwelling men aged ≥65 y without bilateral hip replacements	903	84.2 ± 4	Single dose of 30 mg	Pre-dose urine sample collected: Not specified<br><br>Post-dose urine sample collected: fasting, morning urine sample (not	DXA (QDR 4500W, Hologic Inc.)

Author, year	Location	Population	Sample size (n)	Mean age ± SD (y)	D3-Cr dose	Protocol for urine collection	Additional body composition technique
						the first void), 72–144h post-dose.	
**Zanker et al. 2020 (10)**	United States	Ambulatory community-dwelling men aged ≥65 y without bilateral hip replacements	1,098	83.7 ± 3.7	Single dose of 30 mg	Pre-dose urine sample collected: not specified<br><br>Post-dose urine sample collected: at fasting, 72–144 h post-dose	Not assessed
**Cawthon et al. 2021 (11)**	United States	Ambulatory community-dwelling men aged	1,425	N/A[d]	Single dose of 30 mg	Pre-dose urine sample collected: not specified	Not assessed

Author, year	Location	Population	Sample size (n)	Mean age ± SD (y)	D3-Cr dose	Protocol for urine collection	Additional body composition technique
		≥65 y without bilateral hip replacements				Post-dose urine sample collected: at fasting, morning urine sample, 72–144h post-dose	
**Cegielski et al. 2021 (12)**	United Kingdom	Healthy older males with BMI <35 kg/m^2 and no steroid treatment within 6 months	10	71 ± 4	Single dose of 30 mg	Pre-dose urine sample collected: yes<br><br>Post-dose urine sample collected: a single urine	DXA (equipment specification N/A)

Author, year	Location	Population	Sample size (n)	Mean age ± SD (y)	D3-Cr dose	Protocol for urine collection	Additional body composition technique
						sample at 48h and 72h plus total 24h urine post-dose	
**Zhu et al. 2021 (13)**	United States	Community-dwelling postmenopausal women	73	82.3 ± 5.4	Single dose of 30 mg	Pre-dose urine sample collected: not specified<br><br>Post-dose urine sample collected: at fasting, morning urine sample, 72–144h post-dose	DXA (QDR 4500W, Hologic Inc.)

Author, year	Location	Population	Sample size (n)	Mean age ± SD (y)	D3-Cr dose	Protocol for urine collection	Additional body composition technique
**Zanker et al. 2022 (14)**	United States	Ambulatory community-dwelling men aged ≥65 y without bilateral hip replacements	1,345	84.1 ± 4	Single dose of 30 mg	Pre-dose urine sample collected: not specified<br><br>Post-dose urine sample collected: at fasting, morning urine sample 72–144 h post-dose	DXA (Hologic 4500 scanners, Waltham, MA)
**Cawthon et al. 2022 (15)**	United States	Ambulatory community-dwelling men aged ≥65 y without	1,363	84.2[e]	Single dose of 30 mg	Pre-dose urine sample collected: not specified	Not assessed

Author, year	Location	Population	Sample size (n)	Mean age ± SD (y)	D3-Cr dose	Protocol for urine collection	Additional body composition technique
		bilateral hip replacements				Post-dose urine sample collected: at fasting, morning urine sample, 72–144 h post-dose	
**Orwoll et al. 2022 (16)**	United States	Ambulatory community-dwelling men aged ≥65 y without bilateral hip replacements	1,017	84.2 ± 4	Single dose of 30 mg[c]	Pre-dose urine sample collected: not specified<br><br>Post-dose urine sample collected: at fasting, morning	Not assessed

Author, year	Location	Population	Sample size (n)	Mean age ± SD (y)	D3-Cr dose	Protocol for urine collection	Additional body composition technique
						urine sample, 72–144 h post-dose	
**Cegielski et al. 2022 (17)**	United Kingdom	Ambulatory community-dwelling men aged ≥65 y without bilateral hip replacements	37	72 ± 5	Single dose of 30 mg	Pre-dose urine sample collected: not specified<br><br>Post-dose urine sample collected: over 24h, and spot sample at 48h post-dose	DXA (equipment specification N/A)<br><br>Ultrasound (ImageJ 1.42q software, National Institutes of Health, Bethesda, MD)

Author, year	Location	Population	Sample size (n)	Mean age ± SD (y)	D3-Cr dose	Protocol for urine collection	Additional body composition technique
**Marron et al. 2022 (18)**	United States	Ambulatory community-dwelling men aged ≥65 y without bilateral hip replacements	463	84[e]	Single dose of 30 mg	Pre-dose urine sample collected: not specified<br><br>Post-dose urine sample collected: after 72–144 h post-dose	Not assessed
**Sagayama et al. 2023a (19)**	Japan	Healthy and active males aged ≥18 y that belonged either to a college sports club or an	29 (13 in the endurance group; 9 in the combat group; and 7 in the	Total: 19.9 ± 1.8<br><br>Endurance: 20.6 ± 2.0	Single dose of 30 mg	Pre-dose urine sample collected: not specified	MRI (MAGNETOM Skyra; Siemens, Erlangen, Germany;

Author, year	Location	Population	Sample size (n)	Mean age ± SD (y)	D3-Cr dose	Protocol for urine collection	Additional body composition technique
		athletic organization (judo, triathlon, wrestling, orienteering, and cycling), participated in recreational sports, and/or participated in training during the weekend	active young group)	Combat: 18.9 ± 0.8<br><br>Active young: 20.0 ± 2.0		Post-dose urine sample collected: after overnight fasting (~110h) post-dose, in individual 4.5mL Nunc CryoTubes (Thermo Fisher Scientific, Waltham, MA, USA)	Attractive Basic 3D, PixSpace Ltd. software)<br><br>To build a 4-compartment model:<br>Air displacement plethysmography (BOD POD Tracking System and software

Author, year	Location	Population	Sample size (n)	Mean age ± SD (y)	D3-Cr dose	Protocol for urine collection	Additional body composition technique
							version 4.24.2.4; COSMED, Rome, Italy); DXA (Discovery A, Hologic, Waltham, MA, USA); Deuterium dilution, 0.12 g/kg of 2H2O (99.9%; Taiyo Nippon Sanso, Tokyo, Japan)

Author, year	Location	Population	Sample size (n)	Mean age ± SD (y)	D3-Cr dose	Protocol for urine collection	Additional body composition technique
**Sagayama et al. 2023b (20)**	Japan	Healthy and active males aged ≥18y	28 (13 in the endurance group; 8 in the combat group; and 7 in the active young group)	20.0 ± 2.0	Single dose of 30 mg	Pre-dose urine sample collected: not specified<br><br>Post-dose urine sample collected: overnight fasting (~110h) post-dose, in individual 4.5mL Nunc CryoTubes (Thermo Fisher Scientific,	BIA (logarithmic distribution of 256 frequencies, ranging from 4 to 1000 kHz, SFB7; ImpediMed)

Author, year	Location	Population	Sample size (n)	Mean age ± SD (y)	D3-Cr dose	Protocol for urine collection	Additional body composition technique
						Waltham, MA, USA)	
**Balachandran et al. 2023 (21)**	United States	Men and women ≥70y at high risk for mobility disability	21 (10 in strength training group; and 11 in health education group)	Total: 82.1[e]<br><br>Strength training: 80.4 ± 5.8<br><br>Health education: 83.6 ± 5.4	Single dose of 30 mg	Pre-dose urine sample collected: yes (at week 16, to investigate traces left from baseline dosage)<br><br>Post-dose urine sample collected: fasted, second	DXA (QDR-4500w; Hologic, Waltham, MA)

Author, year	Location	Population	Sample size (n)	Mean age ± SD (y)	D3-Cr dose	Protocol for urine collection	Additional body composition technique
						void, 72–120 h post-dose	
**Beavers et al. 2023 (22)**	United States	Older adults with overweight or obesity	24	68.0 ± 4.4	Single dose of 30 mg	Pre-dose urine sample collected: Not specified<br><br>Post-dose urine sample collected: fasted, not the first void, 72–144 h post-dose	DXA (GE iDXA, Medical Systems, Madison WI)<br>CT (64-slice PET/CT GE Discovery, Medical Systems, Madison WI)

**Author, year**	**Location**	**Population**	**Sample size (n)**	**Mean age ± SD (y)**	**D3-Cr dose**	**Protocol for urine collection**	**Additional body composition technique**
**Cheng et al. 2023 (23)**	United States	Stage II or III colon cancer after curative resection	118	55.2 ± 12.8	Single dose of 60 mg	Pre-dose urine sample collected: Not specified<br><br>Post-dose urine sample collected: fasted, second void, 72–144 h post-dose	CT and DXA not specified

Note. n = number of observations; SD = standard deviation; y = years old; D3-Cr = D3-Creatine; N/A = not available; MRI = magnetic resonance imaging; DXA = dual energy x-ray absorptiometry; BIS = bioimpedance spectroscopy; BIA = multifrequency bioimpedance; CT = computerized tomography.

[a]Mean age ± SD was N/A in the study by Cawthon et al. 2019. Instead, the authors divided their sample by quartiles (Q) of D3-Cr muscle mass/weight. Those in Q1 had lowest amount of muscle mass, while those in Q4 had highest amount of muscle mass. Mean age ± SD Q1: 85.5 ± 4.3 y; Q2: 84.7 ± 4.1 y; Q3: 83.9 ± 4 y; Q4: 82.6 ± 3.2 y.

[b]A value of 20.7 ± 1.9 years (20.6 ± 2 for males and 20.2 ± 1.7 for females) was provided in the study by Morris-Paterson et al.; however, it is not clear if the values refer to mean ± SD

[c]D3-Cr dose reported in the studies by Orwoll et al. (2020 and 2022) was of 30mg; however, the dosage was different from that described in the study protocol they referenced (i.e., Shankaran et al., who used 60mg).

[d]Mean age ± SD was N/A in the study by Cawthon et al. 2021. Instead, the authors divided their sample into two groups: those with lower versus higher D3-Cr muscle mass. Those in the "lower muscle mass" group had a mean age ± SD of 85.1 ± 4.2 y, while those in the "higher muscle mass" category had a mean age ± SD of 83.3 ± 3.7 y.

[e]SD was N/A in the studies by Cawthon et al. 2022, Marron et al. 2022, and Balachandran et al. 2023.

eTable 3. Summary of main findings from studies included in this review comparing D3-creatine dilution with other body composition techniques to measure muscle mass and/or its correspondent compartment as well as its ability to associate with clinical outcomes

**Author, year**	**Main outcomes**		
	**D3-Cr compared with other body composition techniques**	**D3-Cr ability to predict sarcopenia-related outcomes**	**D3-Cr ability to predict other clinical outcomes**
**Clark et al. 2014 (1)**	D3-Cr eMM correlated with MRI MM (r=0.868, p<0.0001)<br>Bland-Altman analysis showed a good agreement (bias=0.72kg; LOA: -5.33 to 6.77kg)<br><br>D3-Cr eMM correlated with DXA LST (r=0.745, p<0.0001)	N/A[a]	N/A[a]

Author, year	Main outcomes<br>D3-Cr compared with other body composition techniques	D3-Cr ability to predict sarcopenia-related outcomes	D3-Cr ability to predict other clinical outcomes
**Buehring et al. 2018 (2)**	D3-Cr eMM correlated with DXA LST (head LST excluded from analysis) (r=0.60 p<0.0001)<br><br>No information provided comparing D3-Cr eMM to BIS FFM	D3-Cr eMM correlated with jump power (r=0.582, 95%CI: 0.44/0.69, p<0.0001) and HGS: (r=0.352, 95%CI: 0.18/0.50, p<0.0001); no correlation (p>0.05) with maximum gait speed (4m) (r=−0.011, 95%CI: −0.20/0.17), SPPB score (r=−0.027, 95%CI: −0.21/0.16); repeat chair raise (r=0.071, 95%CI: −0.12/0.26) , and TUG (r=0.082, 95%CI: −0.11/0.26)	N/A[a]
**Clark et al. 2018 (3)**	In three different methodological approaches, D3-Cr eMM correlated with MRI MM (method 1, steady-state enrichment: r=0.888; method 2, corrected	N/A[a]	N/A[a]

Author, year	Main outcomes		
	D3-Cr compared with other body composition techniques	D3-Cr ability to predict sarcopenia-related outcomes	D3-Cr ability to predict other clinical outcomes
	for observed D3-Cr excretion: r=0.913; method 3, corrected for predicted D3-Cr excretion: r=0.884; p<0.0001) Similar results were found in Bland-Altman analysis for the three methodological approaches assessing D3-Cr eMM (method 1: bias=2.93, LOA= -3.60 to 8.11 kg; method 2: bias=2.91, LOA= -2.36 to 8.17 kg; method 3: bias=3.00, LOA= -2.50 to 8.51)		
**Shankaran et al. 2018 (4)**	D3-Cr eMM correlated with BIS FFM when urine spillage was corrected either by algorithm (r=0.886, p<0.0001) or by	N/A[a]	N/A[a]

Author, year	Main outcomes<br>D3-Cr compared with other body composition techniques	D3-Cr ability to predict sarcopenia-related outcomes	D3-Cr ability to predict other clinical outcomes
	measured 3-day D3-Cr losses (r=0.893, p<0.0001)<br><br>D3-Cr eMM correlated with 24-h Crn excretion eMM when corrected by algorithm (r=0.858, p<0.0001)<br>Bland-Altman analysis showed a good agreement (bias=0.9kg; LOA: -10.53 to 12.38kg)		
**Cawthon et al. 2019 (5)**	D3-Cr eMM correlated with LST (r=0.66 p<0.001), ALST (r=0.68, p<0.001); ALST/height^2 (r=0.55, p<0.001),	Those in lower Q of D3-Cr eMM/weight were more likely not to complete repeat chair stands, the 400m walk, or the balance	Lower D3-Cr eMM/weight related to prevalent mobility limitation (OR=6.14, 95% CI: 3.68/10.27, p<0.001), difficulty bathing/showering (OR=2.64, 95% CI:

Author, year	Main outcomes		
	**D3-Cr compared with other body composition techniques**	**D3-Cr ability to predict sarcopenia-related outcomes**	**D3-Cr ability to predict other clinical outcomes**
	ALST/BMI (r=0.34, p<0.001), and ALST/weight (r=0.13, p<0.001)<br><br>D3-Cr eMM/weight correlated with ALST/BMI (r=0.43 p<0.001), ALST/weight (r=0.60, p<0.001), and LST (r=0.10, p<0.001); no correlation with ALST/height^2 (r=0.02, p=0.355) and ALST (r=0.10, p=0.612)	component of the SPPB compared to those in higher Q (p<0.001)<br>Men in the highest Q of D3-Cr eMM/weight had higher walking speed (p<0.001), greater HGS(p=0.002) and better lower extremity muscle power and force (p<0.001)	1.02/6.82, p=0.033), difficulty getting in/out bed or chairs (OR=3.74, 95% CI: 2.09/6.49, p<0.001), difficulty with heavy housework (OR=4.12, 95% CI: 2.41/7.04, p<0.0001) but not with difficulty lifting/carrying 10lbs (OR=1.63, 95% CI: 0.70/3.79, p=0.061).<br>D3-Cr eMM associated with prevalent mobility limitation (p=0.03), carrying 10 lbs (p=0.043) and heavy housework (p<0.001) but not with difficulty bathing/showering (p=0.633) or difficulty getting out of bed/chairs (p=0.274)

Author, year	Main outcomes		
	D3-Cr compared with other body composition techniques	D3-Cr ability to predict sarcopenia-related outcomes	D3-Cr ability to predict other clinical outcomes
			Those in the lowest Q of D3-Cr eMM/weight were more than twice as likely to report incident serious injurious falls (OR=2.15, 95% CI: 1.74/4.54, p=0.022) and incident mobility limitations (OR=2.15, 95% CI: 1.42 /3.26, p<0.001) compared to those in the highest Q.
**Duchowny et al. 2020 (6)**	Changes in D3-Cr eMM correlated with changes in ALST (r=0.58, p<0.001), ALST/height^2 (r=0.57, p<0.001), DXA LST (r=0.50, p<0.001); no correlation with changes in ALST/weight (r=0.03, p=0.873)	D3-Cr eMM/weight changes correlated with changes in walking speed (r=0.33, p=0.038)<br><br>D3-Cr eMM changes did not correlate with changes in walking speed (r=0.29, p=0.069)	N/A[a]

Author, year	Main outcomes		
	**D3-Cr compared with other body composition techniques**	**D3-Cr ability to predict sarcopenia-related outcomes**	**D3-Cr ability to predict other clinical outcomes**
	Changes in D3-Cr eMM/weight moderately correlated with DXA ALST/height^2 (r=0.46, p=0.003), changes in ALST (r=0.44, p=0.004), and ALST/weight (r=0.36, p=0.024); no correlation with changes in LST (r=0.24, p=0.136)	D3-Cr eMM changes and D3-Cr eMM/weight did not correlate with changes in HGS (r=0.20, p=0.209, and r=0.19, p=0.241)	
**Morris-Patterson et al. 2020 (7)**	D3-Cr eMM correlated with MRI MM (r=0.90, p<0.0001)<br><br>When the creatine pool size was assumed to be 4.3g/kg, Bland-Altman analysis showed D3-Cr overestimated MM	N/A[a]	N/A[a]

Author, year	Main outcomes<br>D3-Cr compared with other body composition techniques	D3-Cr ability to predict sarcopenia-related outcomes	D3-Cr ability to predict other clinical outcomes
	compared to MRI (bias=7.05kg; LOA= -0.62 and 13.47kg). However, differences were no longer observed when the creatine pool size was assumed to be 5.1g/kg (bias=0.25kg; LOA= -5.35 to 5.85kg)		
**Orwoll et al. 2020 (8)**	D3-Cr eMM correlated with DXA LST (r=0.68, p<0.001)[b] but considerable variation between methods was found (R^2=0.46)	Low D3-Cr eMM strongly associated with poor physical performance. Each 1 SD decrement in D3-Cr eMM/weight associated with lower walking speed (B=-0.10 meters/second, 95% CI: -0.11/ -0.09)[c] and shorter chair stand time (B= -0.34/10 seconds, 95% CI: -0.39/ -0.28)[c]	In models adjusted for age, those in the lowest Q of D3-Cr eMM/weight were 9.8-fold (OR; 95% CI: 6.1/15.7, p<0.001) more likely to have a prevalent mobility limitation, and 2.7-fold (OR; 95% CI: 1.8/4.0, p<0.001) more likely to experience incident mobility limitation, and 2.7-fold (OR; 95% CI: 1.5/4.7, p=0.003) more likely to experience an incident injurious fall

Author, year	Main outcomes		
	**D3-Cr compared with other body composition techniques**	**D3-Cr ability to predict sarcopenia-related outcomes**	**D3-Cr ability to predict other clinical outcomes**
**Rogers-Soeder et al. 2020 (9)**	Those with higher D3-Cr eMM by Q had greater DXA ALST compared to those with lower D3-Cr muscle Q (p<0.001)	N/A[a]	Those in higher Q of D3-Cr eMM were more likely to be more physically active compared to those in lower Q (p<0.001)
**Zanker et al. 2020 (10)**	N/A[a]	N/A[a]	D3-Cr eMM and D3-Cr eMM/weight were lower in those who developed incident mobility disability (p<0.001)<br><br>D3-Cr eMM/weight was a relevant predictor for the outcome of incident mobility disability analysis, explaining 35% of variation when walking speed was not included in the analysis

Author, year	Main outcomes		
	**D3-Cr compared with other body composition techniques**	**D3-Cr ability to predict sarcopenia-related outcomes**	**D3-Cr ability to predict other clinical outcomes**
**Cawthon et al. 2021 (11)**	N/A[a]	Those with lower D3-Cr eMM had lower gait speed (p<0.001), number of chair stands in 10 seconds (p<0.001), and HGS (p<0.001) compared to those with higher D3-Cr muscle	Considering all-cause mortality, a higher percentage of deaths was found in those in the lowest Q of D3-Cr eMM (Q1=26%; Q2=17.6%; Q3=7.2%, Q4=5.6%; p<0.001)<br><br>After adjusting for confounding variables, the RR for all-cause mortality across Q remained elevated (Q1 versus Q4=3.4, 95% CI: 1.8/6.7, p<0.001)<br><br>Each SD decrement in D3-Cr eMM/weight associated with 1.9-fold (RR; 95% CI: 1.2/3.1)[c], increased risk of self-reported incident activity

Author, year	Main outcomes<br>D3-Cr compared with other body composition techniques	D3-Cr ability to predict sarcopenia-related outcomes	D3-Cr ability to predict other clinical outcomes
			daily living disability; 1.8-fold (RR; 95% CI: 1.5/2.2)[c] increased risk of self-reported incident mobility disability; and 1.5-fold (RR; 95% CI: 1.3/1.9)[c] increased risk of self-reported incident instrumental activity daily living disability
**Cegielski et al. 2021 (12)**	D3-Cr eMM moderate correlated with ALST (r=0.69, p=0.027); no significant correlation with DXA LST (r=0.55, p=0.098)	N/A[a]	N/A[a]
**Zhu et al. 2021 (13)**	D3-Cr eMM correlated with DXA LST (r=0.50)[c] and ALST (r=0.50)[c]	In logistic regression adjusted for age D3-Cr eMM/weight was associated with SPPB score (OR=5.93, 95% CI: 1.91/18.43)[c] and	N/A[a]

Author, year	Main outcomes<br>D3-Cr compared with other body composition techniques	D3-Cr ability to predict sarcopenia-related outcomes	D3-Cr ability to predict other clinical outcomes
	D3-Cr eMM correlated with DXA LST and ALST when they were adjusted by weight (r=0.62 and 0.58, respectively)[c], BMI (r=0.65 and 0.61, respectively)[c], and height^2 (r=0.41 for both)[c]	physical function assessed by RAND-36 (OR=3.15, 95% CI: 1.13/ 8.84)[c]<br><br>Grip strength was nearly identical in low vs high D3-Cr eMM/weight (21.1 ± 5.02 vs 21.6 ± 5.08)[c]	
**Zanker et al. 2022 (14)**	D3-Cr eMM/weight correlated with ALST/weight (r=0.60, p<0.001); ALST/BMI (r=0.44, p<0.001); pQCT calf muscle area (r=0.28, p<0.001); and no correlation with ALST/height^2 (r=0.02, p>0.05)	D3-Cr eMM/weight correlated with walking speed (r=0.40, p<0.001) and chair stands (r=0.44, p<0.001), HGS (r=0.24, p<0.001), calf intramuscular adipose tissue (r= -0.32, p<0.001), and calf muscle density (r=0.47, p<0.001)	N/A[a]

Author, year	Main outcomes		
	D3-Cr compared with other body composition techniques	D3-Cr ability to predict sarcopenia-related outcomes	D3-Cr ability to predict other clinical outcomes
**Cawthon et al. 2022 (15)**	N/A[a]	N/A[a]	Men who suffered an incident hip fracture had lower D3-Cr eMM/weight (no fracture=0.31 ± 0.05; fracture=0.28 ± 0.04, p=0.004)<br>The incidence of fracture was highest in the lower Q (Q1) of D3-Cr eMM/weight, and the lowest fracture rates were seen in the highest Q (Q4) of D3-Cr eMM/weight. Each SD decrement in D3-Cr eMM/weight was associated with a 1.8-fold higher risk of hip fracture (HR=1.75, 95% CI: 1.22/2.50, p=0.003); a 1.4-fold higher risk of major osteoporotic fracture (HR=1.37, 95% CI: 1.08/1.74, p=0.005); a 1.2-fold higher risk of non-spine fracture (HR=1.21, 95% CI:

Author, year	Main outcomes		
	**D3-Cr compared with other body composition techniques**	**D3-Cr ability to predict sarcopenia-related outcomes**	**D3-Cr ability to predict other clinical outcomes**
			1.02/1.43, p=0.045); and a 1.2-fold higher risk of any clinical fracture (HR=1.22, 95% CI: 1.04/1.43, p=0.018)
**Orwoll et al. 2022 (16)**	N/A[a]	In linear regression model adjusted for age, clinic site, height, and weight D3-Cr eMM was associated with walking speed (B=0.08, 95% CI: 0.06/0.09, p<0.001), chair stands (B=0.72, 95% CI: 0.60/0.84, p<0.001), and HGS (B=2.28, 95% CI: 1.77/2.79, p<0.001) D3-Cr eMM correlated with tibial muscle density assessed by pQCT (r=0.34, p<0.001)	In linear regression model adjusted for age, clinic site, height, and weight D3-Cr eMM was associated with all-cause mortality (B=0.68, 95% CI: 0.55/0.80, p<0.001), mobility limitation (B=0.76, 95% CI: 0.62/0.93, p<0.001), and mobility disability (B=0.54, 95% CI: 0.40/0.72, p<0.001)

Author, year	Main outcomes<br>D3-Cr compared with other body composition techniques	D3-Cr ability to predict sarcopenia-related outcomes	D3-Cr ability to predict other clinical outcomes
**Cegielski et al., 2022 (17)**	D3-Cr eMM correlated with LST (r=0.871, p<0.001), ALST (r=0.896, p<0.001), and vastus lateralis thickness assessed by ultrasound (r=0.36)[c]	D3-Cr eMM was correlated with gait speed (r= -0.12)[c].<br>In multivariate linear regression D3-Cr eMM was associated with HGS (OR=1.34, RMSE=0.25, p<0.001), unilateral leg extension one-repetition maximum (OR=1.64, RMSE=0.35, p<0.001), and leg extension maximum voluntary contraction (OR=6.53, RMSE=1.38, p<0.001)	N/A[a]
**Marron et al., 2022 (18)**	N/A[a]	D3-Cr eMM and D3-Cr eMM/weight were different (p<0.0001) between terciles of gait speed.	N/A[a]

Author, year	Main outcomes		
	D3-Cr compared with other body composition techniques	D3-Cr ability to predict sarcopenia-related outcomes	D3-Cr ability to predict other clinical outcomes
		D3-Cr eMM was associated with gait speed (B= -0.30, SE=0.10, p=0.003), and SPPB (B=0.33, SE=0.04, p<0.001)	
**Sagayama et al. 2023a (19)**	D3-Cr eMM was not different from MRI MM when the creatine pool size was assumed to be 5.1g/kg (p=0.96). However, differences were observed when the creatine pool size was assumed to be 4.3g/kg (p<0.001)<br><br>D3-Cr eMM significantly correlated to MRI MM for both assumptions (r = 0.840,	N/A[a]	N/A[a]

Author, year	Main outcomes<br>D3-Cr compared with other body composition techniques	D3-Cr ability to predict sarcopenia-related outcomes	D3-Cr ability to predict other clinical outcomes
	p<0.001) and the 4-compartment model FFM (r=0.859, p<0.05)<br><br>Bland-Altman analysis showed that D3-Cr overestimated MM compared to MRI (bias=5.1kg; LOA: -2.8 to 13.0kg)[b]		
**Sagayama et al. 2023b (20)**	D3-Cr eMM was not different from BIA eMM (p = 0.102). D3-Cr eMM correlated with BIA eMM (r = 0.751, p<0.01).	N/A[a]	N/A[a]
**Balachandran et al. 2023 (21)**	D3-Cr eMM correlated with baseline DXA ALST (r = 0.79, p<0.001) and LST (r = 0.79, p<0.001). D3-Cr eMM change did not correlate with DXA ALST change (r =	N/A[a]	N/A[a]

Author, year	Main outcomes		
	**D3-Cr compared with other body composition techniques**	**D3-Cr ability to predict sarcopenia-related outcomes**	**D3-Cr ability to predict other clinical outcomes**
	0.19, p=0.49) and LST change (r = 0.40, p=0.13)		
**Beavers et al. 2023 (22)**	D3-Cr eMM change correlated with DXA ALST change (r = 0.46, p=0.03), but did not correlate with DXA LST change (r = 0.15, p=0.50). Stronger correlations were observed in women than in men (LST: women r = 0.35, men r = -0.04; ALST: women r = 0.65, men r = 0.13)[c]<br>D3-Cr eMM change did not correlate with CT skeletal muscle area change in mid-tight (r = 0.16, p=0.46) and trunk (r = 0.17, p = 0.45)	Change in D3-Cr eMM did not correlated with gait speed change (r = 0.15, p=0.50), stair climb change (r = -0.25, p=0.26), knee extension strength change (r = 0.35, p=0.16), and HGS (r = 0.19, p=0.39)	N/A[a]

Author, year	Main outcomes<br>D3-Cr compared with other body composition techniques	D3-Cr ability to predict sarcopenia-related outcomes	D3-Cr ability to predict other clinical outcomes
**Cheng et al. 2023 (23)**	N/A[a]	N/A[a]	Risk of reduced relative dose intensity had no association with SD increase in D3Cr muscle mass [OR: 0.80 (0.49–1.30)], but each SD increase in CT skeletal muscle area [OR: 0.56 (0.38–0.81)] or DXA ALST [0.56 (0.37–0.84)] decreased the risk.<br>More muscle did not decrease the risk of adverse events for D3-Cr eMM [RR: −1.5% (−18.5%, 19.1%)], CT skeletal muscle area [OR: 9.7% (-8.2%, 31.1%)], and DXA ALST [OR: 9.0% (-7.0%, 27.8%)].

Note. n = number of observations; SD = standard deviation; D3-Cr = D3-Creatine; eMM = estimated muscle mass; MRI = magnetic resonance imaging; DXA = dual energy X-ray absorptiometry; LST = lean soft tissue; BIS = bioimpedance spectroscopy; FFM = fat-free mass; ALST = appendicular lean soft tissue; ASMI = appendicular skeletal muscle index; N/A = not available; Q = quartile; LOA

= limits of agreement; SPPB score = short physical performance battery; TUG = time up and go; HGS = hand grip strength; CI = confidence interval; OR = odds ratio; B=beta coefficient; RR = relative risk; pQCT = peripheral quantitative computed tomography; HR = hazard ratio; RMSE = root mean squared error; SE = standard error; BIA = multifrequency bioimpedance; CT = computerized tomography.

[a]Analysis was not performed to compare D3-Cr against another body composition technique or to investigate its ability to predicting health-related outcomes.

[b]Additional data was provided by the author upon an e-mail request.

[c]p value not provided in the study.

REFERENCES

1. Clark RV, Walker AC, O'Connor-Semmes RL, et al. Total body skeletal muscle mass: estimation by creatine (methyl-d3) dilution in humans. J Appl Physiol (1985). 2014;(12):1605-1613. doi: 10.1152/japplphysiol.00045.2014.

2. Buehring B, Siglinsky E, Krueger D, et al. Comparison of muscle/lean mass measurement methods: correlation with functional and biochemical testing. Osteoporos Int. 2018;(3):675-683. doi: 10.1007/s00198-017-4315-6.

3. Clark RV, Walker AC, Miller RR, O'Connor-Semmes RL, Ravussin E, Cefalu WT. Creatine (methyl-D3) dilution in urine for estimation of total body skeletal muscle mass: accuracy and variability vs. MRI and DXA. J Appl Physiol (1985). 2018;(1):1-9. doi: 10.1152/japplphysiol.00455.2016.

4. Shankaran M, Czerwieniec G, Fessler C, et al. Dilution of oral D(3)-Creatine to measure creatine pool size and estimate skeletal muscle mass: development of a correction algorithm. J Cachexia Sarcopenia Muscle. 2018;(3):540-546. doi: 10.1002/jcsm.12278.

5. Cawthon PM, Orwoll ES, Peters K, et al. Strong Relation Between Muscle Mass Determined by D3-creatine Dilution, Physical Performance, and Incidence of Falls and Mobility Limitations in a Prospective Cohort of Older Men. J Gerontol A Biol Sci Med Sci. 2019;(6):844-852. doi: 10.1093/gerona/gly129.

6. Duchowny KA, Peters KE, Cummings SR, et al. Association of change in muscle mass assessed by D3-creatine dilution with changes in grip strength and walking speed. J Cachexia Sarcopenia Muscle. 2020;(1):55-61. doi: 10.1002/jcsm.12494.

7. Morris-Paterson TE, Stimpson SA, Miller RR, et al. Total body skeletal muscle mass estimated by magnetic resonance imaging and creatine (methyl-d_3) dilution in athletes. Scand J Med Sci Sports. 2020;(3):421-428. doi: 10.1111/sms.13585.

8. Orwoll ES, Peters KE, Hellerstein M, Cummings SR, Evans WJ, Cawthon PM. The Importance of Muscle Versus Fat Mass in Sarcopenic Obesity: A Re-evaluation Using D3-Creatine Muscle Mass Versus DXA Lean Mass Measurements. J Gerontol A Biol Sci Med Sci. 2020;(7):1362-1368. doi: 10.1093/gerona/glaa064.

9. Rogers-Soeder TS, Peters KE, Lane NE, et al. Dietary Intake, D3Cr Muscle Mass, and Appendicular Lean Mass in a Cohort of Older Men. J Gerontol A Biol Sci Med Sci. 2020;(7):1353-1361. doi: 10.1093/gerona/glz145.

10. Zanker J, Patel S, Blackwell T, et al. Walking Speed and Muscle Mass Estimated by the D_3-Creatine Dilution Method Are Important Components of Sarcopenia Associated With Incident Mobility Disability in Older Men: A Classification and Regression Tree Analysis. J Am Med Dir Assoc. 2020;(12):1997-2002.e1. doi: 10.1016/j.jamda.2020.03.017.

11. Cawthon PM, Blackwell T, Cummings SR, et al. Muscle Mass Assessed by the D3-Creatine Dilution Method and Incident Self-reported Disability and Mortality in a Prospective Observational Study of Community-Dwelling Older Men. J Gerontol A Biol Sci Med Sci. 2021;(1):123-130. doi: 10.1093/gerona/glaa111.

12. Cegielski J, Wilkinson DJ, Brook MS, et al. Combined in vivo muscle mass, muscle protein synthesis and muscle protein breakdown measurement: a 'Combined Oral Stable Isotope Assessment of Muscle (COSIAM)' approach. Geroscience. 2021;(6):2653-2665. doi: 10.1007/s11357-021-00386-2.

13. Zhu K, Wactawski-Wende J, Ochs-Balcom HM, et al. The Association of Muscle Mass Measured by D3-Creatine Dilution Method With Dual-Energy X-Ray Absorptiometry and Physical Function in Postmenopausal Women. J Gerontol A Biol Sci Med Sci. 2021;(9):1591-1599. doi: 10.1093/gerona/glab020.

14. Zanker J, Blackwell T, Patel S, et al. Factor analysis to determine relative contributions of strength, physical performance, body composition and muscle mass to disability and mobility disability outcomes in older men. Exp Gerontol. 2022;(161):111714. doi: 10.1016/j.exger.2022.111714.

15. Cawthon PM, Peters KE, Cummings SR, et al. Association Between Muscle Mass Determined by D_3-Creatine Dilution and Incident Fractures in a Prospective Cohort Study of Older Men. J Bone Miner Res. 2022;(7):1213-1220. doi: 10.1002/jbmr.4505.

16. Orwoll ES, Blackwell T, Cummings SR, et al. CT Muscle Density, D3Cr Muscle Mass, and Body Fat Associations With Physical Performance, Mobility Outcomes, and Mortality Risk in Older Men. J Gerontol A Biol Sci Med Sci. 2022;(4):790-799. doi: 10.1093/gerona/glab266.

17. Cegielski J, Brook MS, Phillips BE, et al. The Combined Oral Stable Isotope Assessment of Muscle (COSIAM) reveals D-3 creatine derived muscle mass as a standout cross-sectional biomarker of muscle physiology vitality in older age. Geroscience. 2022;(4):2129-2138. doi: 10.1007/s11357-022-00541-3.

18. Marron MM, Orwoll ES, Cawthon PM, Lane NE, Newman AB, Cauley JA. Oxylipins Associated with D3-Creatine Muscle Mass/Weight and Physical Performance among Community-Dwelling Older Men. Int J Mol Sci. 2022;(21):12857. doi: 10.3390/ijms232112857.

19. Sagayama H, Yamada Y, Kondo E, et al. Skeletal muscle mass can be estimated by creatine (methyl-d3) dilution and is correlated with fat-free mass in active young males. Eur J Clin Nutr. 2023;(3):393-399. doi: 10.1038/s41430-022-01237-9.

20. Sagayama H, Kondo E, Tanabe Y, et al. Comparison of Bioelectrical Impedance Indices for Skeletal Muscle Mass and Intracellular Water Measurements of Physically Active Young Men and Athletes. J Nutr. 2023;(9):2543-2551. doi: 10.1016/j.tjnut.2023.07.010. Epub 2023 Jul 24.

21. Balachandran AT, Evans WJ, Cawthon PM, et al. Comparing D3-Creatine Dilution and Dual-Energy X-ray Absorptiometry Muscle Mass Responses to Strength Training in Low-Functioning Older Adults. J Gerontol A Biol Sci Med Sci. 2023;(9):1591-1596. doi: 10.1093/gerona/glad047.

22. Beavers KM, Avery AE, Shankaran M, et al. Application of the D3-creatine muscle mass assessment tool to a geriatric weight loss trial: A pilot study. Journal of Cachexia, Sarcopenia and Muscle. 2023; (5):2350-2358. doi: 10.1002/jcsm.13322.

23. Cheng E, Caan BJ, Cawthon PM, et al. Body Composition, Relative Dose Intensity, and Adverse Events among Patients with Colon Cancer. Cancer Epidemiol Biomarkers Prev. 2023;(10):1373-1381. doi: 10.1158/1055-9965.EPI-23-0227.
